# A pathway for error-free non-homologous end joining of resected meiotic double-strand breaks

**DOI:** 10.1093/nar/gkaa1205

**Published:** 2021-01-06

**Authors:** Talia Hatkevich, Danny E Miller, Carolyn A Turcotte, Margaret C Miller, Jeff Sekelsky

**Affiliations:** Curriculum in Genetics and Molecular Biology, 120 Mason Farm Road, University of North Carolina, Chapel Hill, NC 27599, USA; Department of Pediatrics, Division of Medical Genetics, University of Washington, Seattle, Washington and Seattle Children's Hospital, Seattle, WA 98105, USA; Curriculum in Genetics and Molecular Biology, 120 Mason Farm Road, University of North Carolina, Chapel Hill, NC 27599, USA; Department of Biology, University of North Carolina, 120 South Road, Chapel Hill, NC 27599, USA; Curriculum in Genetics and Molecular Biology, 120 Mason Farm Road, University of North Carolina, Chapel Hill, NC 27599, USA; Department of Biology, University of North Carolina, 120 South Road, Chapel Hill, NC 27599, USA; Integrative Program in Biological and Genome Sciences, 250 Bell Tower Drive, University of North Carolina, Chapel Hill, NC 27599, USA

## Abstract

Programmed DNA double-strand breaks (DSBs) made during meiosis are repaired by recombination with the homologous chromosome to generate, at selected sites, reciprocal crossovers that are critical for the proper separation of homologs in the first meiotic division. Backup repair processes can compensate when the normal meiotic recombination processes are non-functional. We describe a novel backup repair mechanism that occurs when the homologous chromosome is not available in *Drosophila melanogaster* meiosis. In the presence of a previously described mutation (*Mcm5^A7^*) that disrupts chromosome pairing, DSB repair is initiated by homologous recombination but is completed by non-homologous end joining (NHEJ). Remarkably, this process yields precise repair products. Our results provide support for a recombination intermediate recently proposed in mouse meiosis, in which an oligonucleotide bound to the Spo11 protein that catalyzes DSB formation remains bound after resection. We propose that this oligonucleotide functions as a primer for fill-in synthesis to allow scarless repair by NHEJ. We argue that this is a conserved repair mechanism that is likely to be invoked to overcome occasional challenges in normal meiosis.

## INTRODUCTION

Crossovers promote the accurate segregation of homologous chromosomes during meiosis. The formation of crossovers begins with the introduction of double-strand breaks (DSBs) by the highly conserved endonuclease Spo11 and its associated proteins (reviewed in 1). Meiotic DSB repair differs from mitotic DSB repair in several important ways. First, DSB repair in mitotically proliferating cells can employ several repair strategies, including non-homologous end joining (NHEJ), various homology-directed pathways and DNA polymerase theta-mediated end joining (reviewed in 2). In contrast, DSBs made during meiosis are, under normal circumstances, repaired exclusively by homologous recombination (HR). NHEJ begins with binding of the Ku heterodimer to DNA ends, so it has been suggested that the bound Spo11 enzyme also blocks NHEJ by preventing binding of Ku (3,4). An early step in HR is 5′-to-3′ resection resulting in long, single-stranded 3′ overhangs that also prevent NHEJ (5–8).

A second key difference between mitotic and meiotic DSB repair is that the sister chromatid is typically used as a template for HR during mitotic repair but in meiosis the homologous chromosome is used. Bias for using the homolog is established by the presence of the meiotic chromosome axis and meiosis-specific recombination enzymes (9–11). The axis, made up of cohesins and meiosis-specific proteins, organizes the chromosome into an array of loops which later serve as the base of the synaptonemal complex (SC) that joins homologous chromosomes together (reviewed in 12).

Finally, mitotic HR is regulated to avoid generation of reciprocal crossovers but in meiosis reciprocal crossing over is essential and is actively promoted at selected DSB (reviewed in 13). Complexes specific to meiotic recombination, including the ZMM proteins found in many eukaryotes (reviewed in 14) and the mei-MCM complex of flies (15), block noncrossover pathways and/or promote crossing-over.

When meiotic recombination is disrupted by mutations in key genes, alternative repair pathways are activated to ensure that all DSBs get repaired. If HR is blocked at early steps, NHEJ and other pathways can intervene (5–8,16). If later steps in the meiotic crossover pathway are compromised, mitotic-like HR mechanisms take over (reviewed in 17).

An interesting question is what happens when recombination pathways are intact but a homologous chromosome is not available to use as a template. In budding yeast and *Arabidopsis*, meiosis in haploids or when one chromosome is monosomic reveals that the sister chromatid can be used as a repair template, though recombination progresses differently (18–20).

We describe here a novel pathway for repair of meiotic DSBs when a homolog is not present. Hatkevich *et al.* (21) recently described aberrant meiotic chromosome behavior in *Drosophila melanogaster* females with the *Mcm5^A7^* mutation. Mcm5 is best known as a component of the mini-chromosome maintenance (MCM) complex that is part of the pre-replication initiation complex and is essential for DNA replication (22). *Mcm5^A7^* is a separation-of-function missense mutation that has no detectable defects in replication but has meiosis-specific defects in chromosome behavior. In these mutants, chromosomes pair during leptotene-zygotene, but most pairing is lost by pachytene. Hatkevich *et al.* proposed that this loss of pairing is a result of earlier defects in loading of centromeric cohesins and in centromere clustering. Despite the loss of chromosome pairing, synaptonemal complex appears to be normal, indicating widespread heterosynapsis (SC between non-homologous chromosomes). Previous studies had found that DSBs appear and disappear with approximately normal kinetics in *Mcm5^A7^* mutants (23). The *Mcm5^A7^* mutant therefore provides an opportunity to study the fate of DSBs made in the presence of SC but in the absence of a homolog to use as a template for repair. We show below that, under these conditions, meiotic DSB repair is attempted first by HR but is then completed by NHEJ. Remarkably, whole-genome sequencing fails to find deletions predicted to be produced by NHEJ, suggesting precise repair. We propose a model in which Spo11-bound oligonucleotides annealed to the ends of resected DSBs can function as primers for synthesis to fill in resected regions, allowing error-free repair by NHEJ. Recent studies provide evidence for a similar intermediate in mouse meiosis (24,25), suggesting that this may be a conserved mechanism for repair of meiotic DSBs when the homologous chromosome is unavailable.

## MATERIALS AND METHODS

### Genetic assays

Flies stocks were maintained on standard medium at 25°C. In this article, *Drosophila* nomenclature was generalized for ease of reading. Specific genotypes are listed in the table below.

**Table:** 

Genotype in text	Specific genotype
*WT*	*y w^1118^*
*Mcm5^A7^*	*Mcm5^A7^* / *Df(3R)Exel7305*
*mei-P22*	*mei-P22^103^*
*mei-P22 Mcm5^A7^*	*mei-P22^103^, Mcm5^A7^* / *Df(3R)Exel7305*
*lig4*	*lig4^57^*
*lig4 mei-P22*	*lig4^57^*; *mei-P22^103^*
*lig4 Mcm5^A7^*	*lig4^57^*; *Mcm5^A7^*/ *Df(3R)Exel7305*
*lig4 mei-P22 Mcm5^A7^*	*lig4^57^*; *mei-P22^103^Mcm5^A7^* / *Df(3R)Exel7305*
*spn-A*	*spnA^093A^* / *spn-A^057^*
*Mcm5^A7^* *spn-A*	*Mcm5^A7^* / *Df(3R)Exel7305, spn-A*^093A^/ *spn-A^057^*
*mh*	*mh^1^* / *mh^KG05829^*
*mh Mcm5^A7^*	*mh^1^* / *mh^KG05829^*; *Mcm5^A7^*/ *Df(3R)Exel7305*
*gkt Mcm5^A7^*	*P*{*nos::GAL4*} / *P*{*UAS::gkt RNAi*};*Mcm5^A7^* / *Df(3R)Exel7305*

Fewer than 20% of embryos produced by *Mcm5^A7^* mutant females survive. This is like other meiotic recombination mutants and is attributed to aneuploidy arising from nondisjunction (26). Nondisjunction and recombination were scored in surviving progeny. *X* chromosome NDJ was evaluated by scoring progeny from virgin females of the desired genotype crossed with *y cv v f/T(1:Y)B^S^*males. Viable exceptional *XXY* females have Bar-shaped eyes, and viable exceptional *XO* males have wild-type eye shape and express the *y cv v f* mutant phenotypes. Crossovers on chromosome *2L* were measured by crossing virgin *net dpp^d^^-^^ho^ dpy b pr cn/*+ females of the desired genotype to *net dpp^ho^ dp b pr cn* males. Vials of flies were flipped after three days of mating. Resulting progeny were scored for all phenotypic markers.

### Dissection and immunofluorescence (IF) of whole-mount germaria

In all immunofluorescent and genetic experiments, *Drosophila melanogaster* adult females 3–10 days old were used. In whole-genome sequencing studies, individual male progeny were used. Ten 3- to 5-day-old virgin females were fattened overnight with yeast paste in vials with ∼5 males of any genotype. Ovaries were dissected in fresh 1× PBS and incubated in fixative buffer for 20 min. Fixative buffer: 165 μl of fresh 1× PBS, 10 μl of N-P40, 600 μl of heptane, and 25 μl of 16% formaldehyde. After being washed three times in 1× PBS + 0.1% Tween-20 (PBST), ovaries were incubated for 1 h in 1 ml PBST + 1% BSA (10 ml of PBST + 0.1 g BSA). Ovaries were incubated overnight in 500 μl primary antibody diluted in 1 ml PBST + 1% BSA at 4°C on a rocking nutator. Ovaries were then washed 3× in PBST and incubated in 500 μl secondary antibody diluted at 1:500 in PBST + 1% BSA for 2 h under foil. Ovaries were mounted in 35 μl of ProLong Gold + DAPI on a microscope slide using fine-tip forceps to spread ovaries.

Antibodies for C(3)G (27) and γ-H2Av (Rockland) were used. Images of whole-mount germaria were taken on a Zeiss LSM880 confocal laser scanning microscope using 63×/0.65 NA oil immersion objective, with a 2× zoom using ZEN software. Images were saved as .czi files and processed using FIJI (28). DSBs were quantified as described below.

For quantification of γ-H2Av foci, FIJI (28) was used to visualize images, and contrast and brightness were adjusted for optimal viewing. In each region of the germarium, individual γ-H2Av foci were manually counted in nuclei expressing C(3)G. Data are represented as mean ± 95% confidence intervals. Genotypes were blinded to the scorer.

### Whole-genome sequencing, alignment and SNP calling

*Mcm5^A7^* was crossed into a *y cv v f* line with isogenized chromosomes *X* and *2* (*iso1*). *Df(3R)Exel7305* was crossed into a *w^1118^* line with isogenized chromosomes *X* and *2*. These two lines were crossed to one another and resulting females (heterozygous for *iso1* and *w^1118^* on *X* and *2* and *Mcm5^A7^*/*Df(3R)Exel7305* on *3*) were backcrossed with *iso1* males. Surviving male progeny were used for whole-genome sequencing.

DNA was isolated from individual male progeny using the Qiagen Blood and Tissue Kit and sheared using a Covaris S220 sonicator. KAPA HTP Library Prep kit (KAPA Biosystems, Cat. No. KK8234) was used to construct libraries with NEXTflex DNA barcodes (BiooScientific, Cat. No. NOVA-514104). Libraries were pooled and run on an Illumina NextSeq 500 as 150 bp paired-end samples using the high-output mode. Real Time Analysis software version 2.4.11 and bcl2fastq2 v2.18 demultiplexed reads and generated FASTQ files. FASTQ files were aligned to version 6 of the *Drosophila melanogaster* reference genome (dm6) using bwa mem version 0.7.17-r1188 with default settings (29). SNPs were called using both Samtools (30) and GATK HaplotypeCaller (31). Depth of coverage analysis revealed two X0 males (mcm5-01 and mcm5-12).

### Detection of CO and NCO-GC events and calculation of expected events

CO and NCO-GC events were identified as in Miller *et al.* (32). Briefly, parental stocks were sequenced to identify variants. SNP density per chromosome arm was: 1/531 bp for the *X*, 1/255 bp for *2L*, 1/254 bp for *2R*, 1/249 bp for *3L*, and 1/318 bp for *3R*. For each individual offspring sequenced (*n* = 28), the variant call file (VCF) generated by Samtools and GATK HaplotypeCaller were examined to identify potential differences from parental chromosomes. Each candidate change was examined visually using the Integrative Genomics Viewer (IGV) (33). From the 28 genomes we identified 11 COs and 16 NCO-GCs; 11 NCO-GCs were validated using PCR and Sanger sequencing.

### Identification of deletions and generation of simulated deletions

Multiple approaches were used to search for deletions. First, VCF files generated by Samtools and GATK HaplotypeCaller were searched for unique deletion polymorphisms. Candidate deletions were visually examined using IGV (33). Second, Pindel v0.2.5b9 (34) and Breakdancer v1.1_20100719 (35) were used to identify larger deletions and a custom script was used to parse the output and identify novel events. To estimate the sensitivity of this approach, we randomly selected one 25 Mb region on each of three different chromosome arms from different sequenced progeny. We extracted insertions and deletions from each VCF file, then examined the corresponding region visually using IGV. Together, these regions had 101 predicted indels (mean size six base pairs, range 37 bp deletion to 27 bp insertion). 99 of these were present by IGV (the other two each had a small minority of reads with the indel). Thirteen additional indels not present in the VCF were found, giving an estimated sensitivity of 89% (Supplementary Table S1). For large deletions, we used IGV to examine the left end of the *X* chromosome (starting at position 300,000 to avoid poor mapability in the repeat-rich subtelomeric region) for large deletions. We did this for the *w^1118^* parental chromosome and four progeny flies that inherited this chromosome. Of the first 21 deletions, one was not detected in any of the flies, suggesting it may be a complex rearrangement. Of the other 20, 94 instances across the five *X* chromosome were successfully detected by Pindel, for an estimated sensitivity of 94% (Supplementary Table S2).

As a further test, we conducted simulations as in Miller (36). Briefly, 200 simulated genomes were created based on the dm6 reference genome, 100 with deletions of 1–20 bp, and 100 with deletions varying from 1 to 1000 bp. SNPs were randomly placed approximately 1 every 500 bp in these synthetic genomes. For each genome a minimum of five and maximum of 25 deletions were made. Each deletion was randomly assigned a size, a position on *X*, *2* or *3*, and one of the four haplotypes corresponding to the four meiotic products; one of these haplotypes was then randomly selected as the offspring. A second synthetic genome representing the male haplotype was also generated with no *X* but with a *Y* chromosome, with SNPs randomly placed approximately 1 every 500 bp. Using each synthetic genome as a reference, ART (37) was used to generate 100-bp paired-end reads at ∼10× coverage with an average insert size of 400 bp. FASTQ files were then combined into a single FASTQ and aligned to dm6 using bwa. These synthetic genomes were analyzed as described above for the experimental sequence, with SNPs called using both Samtools and GATK HaplotypeCaller, and Pindel and Breakdancer used to identify larger deletions. Analysis of VCF files from simulated genomes revealed that the approaches described above identified >85% of novel deletions of 1–20 bp and >65% of novel deletions 1–1000 bp.

To calculate the number of deletions we expected to recover we used a conservative assumption of 15 DSBs per meiosis. Based on the whole-genome sequencing data, HR is decreased by ∼80% in *Mcm5^A7^* mutants, suggesting that NHEJ repairs at least 12 DSBs per meiosis; each oocyte receives one of four chromatids, resulting in an average of three deletions per progeny. Therefore, we expect 84 deletions in our sample size of 28 meioses. Based on the simulated genomes described above, we should detect, conservatively, 71 of these if they are small, 48 if they are large.

Normal and exceptional (those resulting from a nondisjunction event) progeny were counted. To adjust for inviable exceptional males and females, viable exceptional class was multiplied by 2. % *X*-NDJ = 100 × ([2 × viable exceptional females] + [2 × viable exceptional males])/corrected total progeny. Statistical comparisons were performed as in Zeng *et al.* (38).

Genetic distances are expressed in centiMorgans (cM), calculated by 100 × (*R*/*n*), where *R* is the number of crossovers in a given interval (including those in single-, double-, and triple-crossover chromosomes), and *n* is the total number of progeny scored. 95% confidence intervals were calculated from variance, as in Stevens (39). Molecular distances (in Mb) are from the positions of genetic markers on the *Drosophila melanogaster* reference genome, release 6.12 (40). Crossover density (or frequency), as calculated by cM/Mb, exclude transposable elements (41).

### Statistical analyses

For number of crossover and noncrossover events detected through whole-genome sequencing, we conducted a one sample *t*-test using the QuickCalcs online calculator (https://www.graphpad.com/quickcalcs/oneSampleT1/). Expected means (events per fly) were from a sample of 196 whole-genome sequences of progeny from parents with similar parental chromosomes *X* and *2* (42); two-tailed *P* values are reported. Numbers of γ-H2Av foci were compared through unpaired *t*-tests between same developmental stages, using Prism for Windows 9.0.0 (GraphPad).

## RESULTS

### Reduction of crossovers and loss of crossover patterning in *Mcm5^A7^* mutants

**Figure 1.**
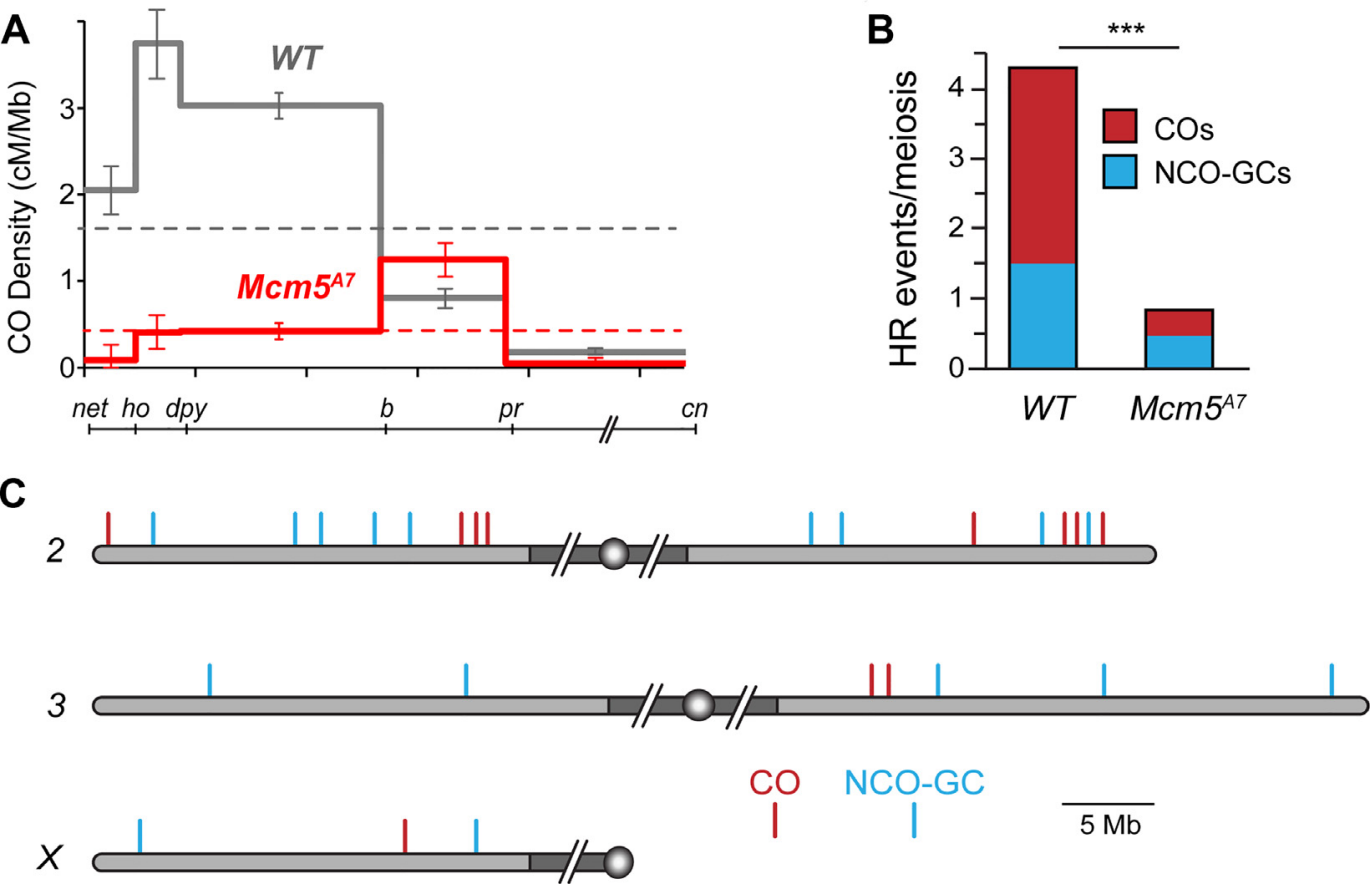
Homologous recombination is severely reduced in *Mcm5^A7^* mutants. (**A**) Crossover density (cM/Mb) across *2L* in wild-type females and *Mcm5^A7^* mutants (*n* = 2070). Dashed lines represent mean crossover density across the region assayed. Error bars indicate 95% confidence intervals. The line below the graph shows the markers used along *2L* and *2R* (slashes represent the centromere and pericentric heterochromatin, which are not included). (**B**) Number of crossovers (COs) and noncrossover gene conversion events (NCO-GCs) per meiosis in wild type (*n* = 196 meioses, dataset from Miller *et al.* (42) and *Mcm5^A7^*mutants (*n* = 28 meioses). ***P < 0.0001, one sample *t*-test, two-tailed. (**C**) COs and NCO-GCs identified by WGS of individual males from *Mcm5^A7^* females. Gray shaded area represents euchromatic sequence, while dark gray shaded area represents the pericentric heterochromatin (not to scale), circles represent centromeres. *2L* is vertically aligned with the graph in panel (A).

*Mcm5^A7^* is a missense, separation-of-function mutation recovered in a screen for mutants with high levels of meiotic nondisjunction (23). The high level of nondisjunction observed in *Mcm5^A7^* mutants was attributed to a 90% reduction in meiotic crossovers on the *X* chromosome. The decrease in crossovers was non-uniform, with the interval spanning the centromere being decreased by only 80%. We extended this analysis to an autosome by scoring crossovers along *2L* and observed an overall decrease of about 75%. However, the nonuniformity was more pronounced as one interval (*b* to *pr*) exhibits 150% of crossovers compared to wild-type flies (Figure 1A, Supplementary Table S3). Notably, in wild-type flies this interval accounts for about 10% of the crossovers on *2L*, while in *Mcm5^A7^* mutants more than half of all crossovers are in this region.

Crossover distribution is a product of meiotic spatial patterning phenomena, especially crossover interference. Interference is observed as a decrease in the frequency of double crossovers from the number expected if crossovers are independent of one another (reviewed in 43). Because crossover distribution is perturbed in the *Mcm5^A7^* mutant, we asked whether interference is affected. Due to the strong reduction in crossovers in the *Mcm5^A7^* mutant, there is only one pair of adjacent intervals in our dataset with enough crossovers to estimate interference: *dp* to *b* and *b* to *pr* (Supplementary Table S1). In wild-type flies, the incidence of double crossovers in these two intervals is less than half the number expected if they are independent (41). In *Mcm5^A7^* mutants, however, six double crossovers were expected and seven were observed, consistent with a partial or complete loss of interference.

The SC has been suggested to be important for achieving interference (43,44). The effect on interference may be explained by the presence of heterosynapsis in *Mcm5^A7^* mutants (21); we hypothesize that discontinuities in the SC (i.e. heterosynapsis to homosynapsis) impede interference. The higher incidence of crossovers in the *b* to *pr* interval is intriguing. In *Mcm5^A7^* mutants, chromosome pairing appears to be normal in late leptotene but is disrupted as SC assembles. We speculate that the higher frequency of crossovers in the *b* to *pr* region might result from greater stability of pairing in this region.

### Homologous recombination is severely reduced in *Mcm5^A7^* mutants

Because the number of meiotic DSBs in *Mcm5^A7^* mutants is normal (23, see below), the reduction in crossovers in *Mcm5^A7^* mutants likely reflects either a direct role for Mcm5 in the crossover pathway or a reduced ability to use the homologous chromosome for HR. In wild-type females, about a third to a quarter of DSBs are repaired as crossovers, with the remainder becoming noncrossovers; only crossovers would be reduced if these mutants have a crossover-specific defect, but both crossovers and noncrossovers would be reduced if the defect is in accessing the homologous chromosome. We used whole-genome sequencing to measure both crossovers and noncrossovers in individual offspring from *Mcm5^A7^* females. Since only one of four chromatids goes into the oocyte, only half of the crossover and one quarter of the noncrossovers will be detected. Only those noncrossovers that are accompanied by a gene conversion tract that spans at least one polymorphism (noncrossover gene conversions; NCO-GCs) will be detected.

We whole-genome sequenced 28 individual progeny from *Mcm5*^A7^ females, identified crossover and NCO-GC events, and compared this data to a previously published dataset of 196 individual progeny from wild-type females (42). Genome-wide, we find an 86% reduction in crossovers (78 expected versus 11 observed, *P* < 0.001 based on a two-tailed, one sample *t*-test) and a 67% reduction in NCO-GCs (42 expected versus 14 observed, *P* < 0.0001) (Figure 1B). A reduction in both crossovers and NCO-GCs is consistent with a general inability to complete interhomolog HR in *Mcm5^A7^* mutants.

Given the results with traditional crossover mapping described above, we also asked whether crossovers and NCO-GCs cluster in the genome. On *2L*, three of the four crossovers observed are located within a 2 Mb span between *b* and *pr*, the region in which genetic experiments also revealed an elevated rate of exchange (Figure 1C). Similarly, on *2R*, three of the four crossovers are grouped within a 2 Mb region, and the two COs on *3R* occur within 0.4 Mb of one another (no COs were recovered on *3L* and only a single CO was recovered on the *X*). In contrast, there is no obvious clustering of NCO-GCs, which appear to be evenly distributed throughout the genome (Figure 1C). These findings suggest that CO and NCO formation may be differentially controlled in *Mcm5^A7^* mutants.

### Most double-strand breaks are repaired by Region 3 in *Mcm5^A7^* mutants

**Figure 2.**
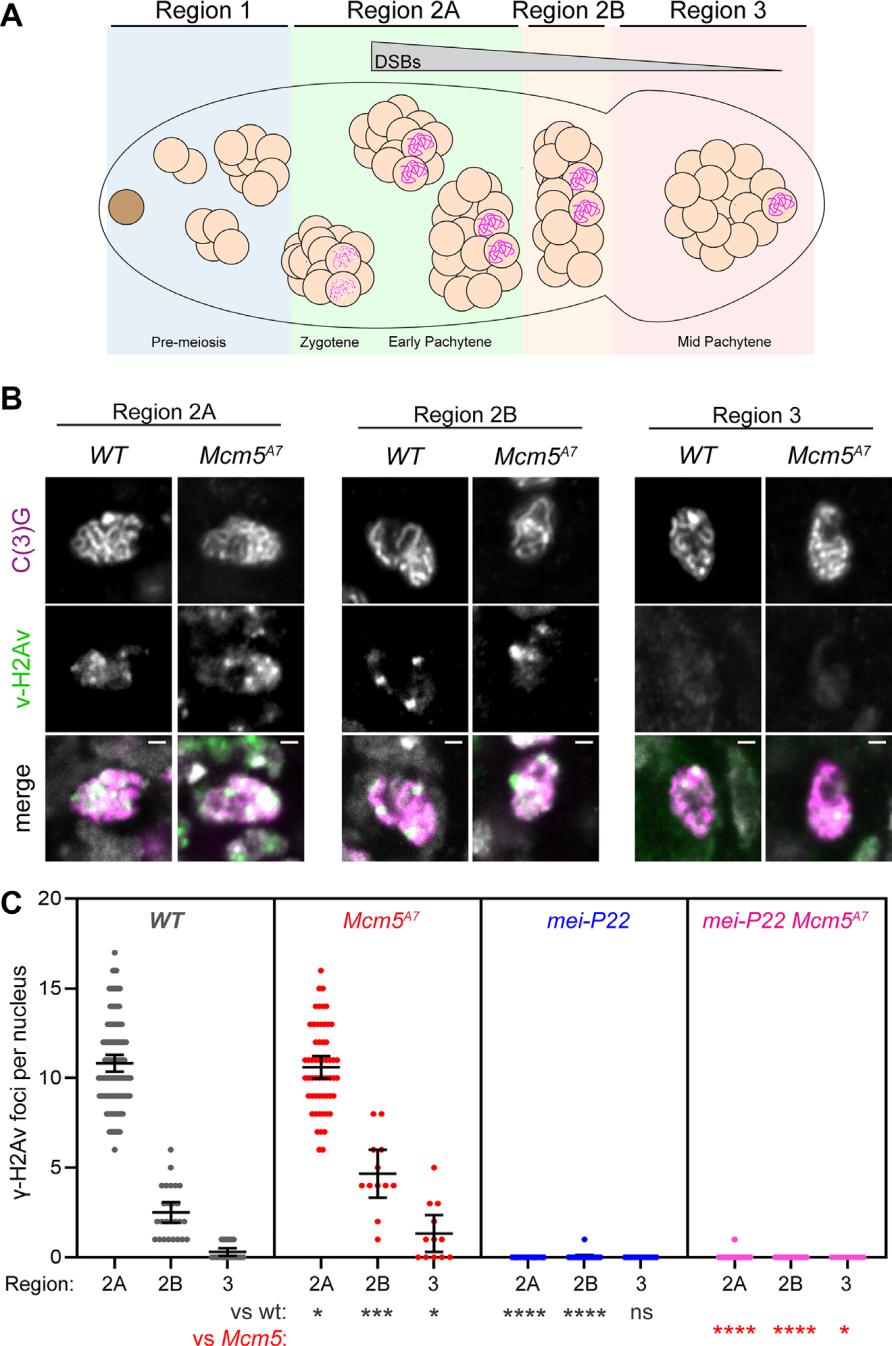
Meiotic double-strand breaks are repaired with normal kinetics in *Mcm5^A7^*. (**A**) Schematic of the *Drosophila* germarium. The germline stem cell (brown), cystoblast (not shown), and developing 2-, 4- and 8-cell cystocytes (cc) reside in the pre-meiotic mitotic region of the germarium, Region 1. Meiotic onset occurs within the most anterior 16-cc in Region 2A, which is cytologically defined as zygotene. In early pachytene (Region 2A), formation of the synaptonemal complex (SC, pink scribble) is complete and meiotic double-strand break (DSB) formation begins. Meiotic DSBs are repaired as the 16-cc progresses through early pachytene (Region 2B) and into mid-pachytene (Region 3). (**B**) Representative images of γ-H2Av foci (green) in *WT* and *Mcm5^A7^*meiotic nuclei (as defined by the present of the SC component, C(3)G, pink) within Regions 2A, 2B, and 3. Merge images include DAPI in gray. Image brightness and contrast have been adjusted for clarity. Bright puncta in merged images are DAPI-dense chromocenters. (**C**) Quantification of γ-H2Av foci. Each dot represents one nucleus; bars show mean and 95% confidence intervals. Results of unpaired *t*-tests compared to wild type (black) and to *Mcm5^A7^* (red) are indicated below. ns, *P* > 0.05; *P < 0.05, **P < 0.01, ***P < 0.001, ****P < 0.0001. *n* = (across the three regions) for *WT*: 99, 25, 18; for *Mcm5^A7^*: 48, 10, 10; for *mei-P22*: 60, 19, 11; for *mei-P22 Mcm5^A7^*: 68, 19, 11.

Lake *et al.* (23) found that meiotic DSBs, as marked by γ-H2Av foci, are created and repaired with normal timing in *Mcm5^A7^*mutants. To extend this result, we counted γ-H2Av foci during DSB formation and repair. Foci first appear in Region 2A of the germarium, then decrease in number as the pro-oocyte progresses through Region 2B and into Region 3, with few or no foci remaining in Region 3, by which time breaks are thought to be repaired (Figure 2A).

The number of foci in Region 2A may underestimate the total number of DSBs per meiotic cell due to asynchrony in formation and repair and inability to resolve foci near one another in 3D space within the compact nucleus. Total DSB estimates range from 11 to 24 based on γ-H2Av foci using different antibodies and whole-genome sequencing (42,45). *Mcm5^A7^*mutants have a nearly normal number of γ-H2Av foci in Region 2A compared to wild type (Figure 2B, C). Given the canonical function of Mcm5 in DNA replication (22), it seemed possible that some DSBs in *Mcm5^A7^* mutants might result from problems during pre-meiotic S phase. To test this, we used a *mei-P22* mutation to eliminate meiotic DSBs. Mei-P22 is required for all meiotic DSBs in *Drosophila* and is thought to be the ortholog of the TopoVIBL non-catalytic subunit of the enzyme that makes meiotic DSBs (46–48). γ-H2Av foci are nearly eliminated in both *mei-P22* single mutants and *mei-P22 Mcm5^A7^* double mutants (Figure 2B, C), indicating that breaks in *Mcm5^A7^*mutants are meiotically-induced.

Although the numbers of foci Regions 2B and 3 are significantly higher in *Mcm5^A7^* mutants than in wild type, most foci are still gone by Region 3 (1.3 in Region 3 versus 10.4 in Region 2A, Figure 2C). Thus, we estimate that at least 88% of DSBs made in *Mcm5^A7^* mutants are repaired by Region 3. Since interhomolog HR is reduced by >80% (Figure 1), these DSBs must be repaired in other ways.

### Repair of most DSBs in *Mcm5^A7^* mutants requires both Rad51 and DNA ligase IV

**Figure 3.**
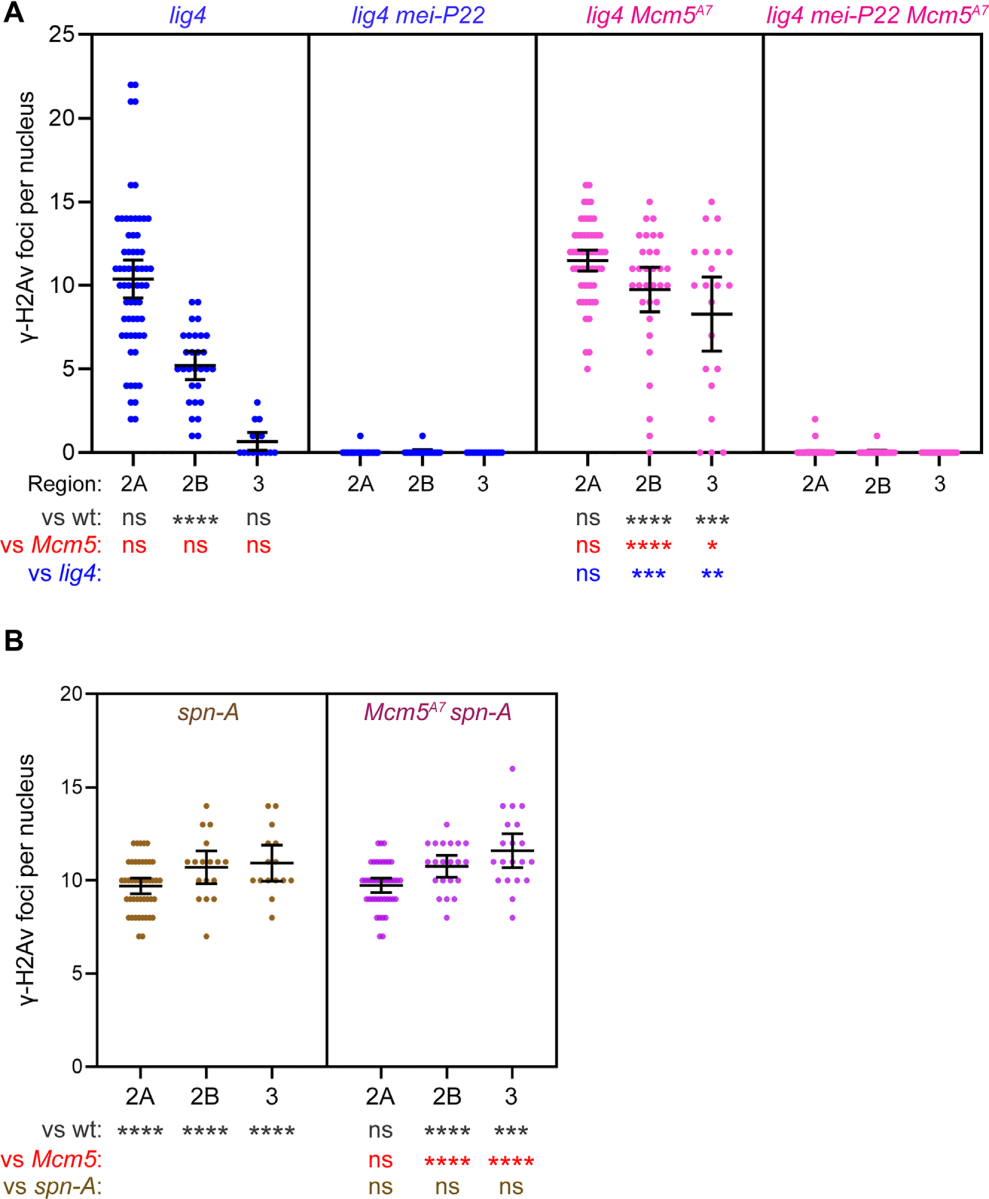
Meiotic DSB repair in *Mcm5^A7^* mutants requires the NHEJ protein Lig4 and the strand exchange protein Spn-A. (**A** and **B**) Quantification of γ-H2Av foci. Each dot represents one nucleus; bars show mean and 95% confidence intervals. Results of unpaired *t*-tests are shown below (see Figure 2 legend). *n* = (across the three regions) for *lig4*: 63, 29, 15; for *lig4 mei-P22*: 46, 17, 14; for *lig4 Mcm5^A7^*: 57, 32, 21; for *lig4 mei-P22 Mcm5^A7^*: 74, 24, 17; for *spn-A*: 44, 17, 15; for *Mcm5^A7^ spn-A*: 42, 21, 20.

Previous studies have shown that *Mcm5^A7^*mutants do not exhibit an increase in inter-sister crossing-over (6,21) suggesting that a strong barrier to use of the sister as an HR repair template is present in regions of heterosynapsis. Together, our data suggest that DSBs made in regions of heterosynapsis are repaired by one or more non-HR pathways. One possibility is NHEJ. NHEJ is normally prevented during meiotic DSB repair but can function when there are defects in HR (5–8,16,49). To determine whether NHEJ is responsible for meiotic break repair in *Mcm5^A7^*mutants, we asked whether repair is dependent on DNA ligase IV (Lig4), an enzyme both specific to and essential for NHEJ (50). In *lig4* single mutants, the γ-H2Av foci in Regions 2A, 2B and 3 are similar to those in wildtype and *Mcm5^A7^* (like *Mcm5^A7^*, there is a small but significantly higher number of foci in Region 2B), with more than 90% of DSBs being repaired by Region 3 (Figure 3A, panel 1), and most γ-H2Av foci in *lig4* mutants are dependent on Mei-P22 (Figure 3A, panel 2). These results confirm previous observations that Lig4 does not have a detectable role in meiotic break repair in *Drosophila* (6). In *lig4 Mcm5^A7^* double mutants, the number of γ-H2Av foci in Region 2A is similar to that seen in wild-type and single mutants, but unlike in single mutants, most foci persist through Region 2B and into Region 3 (Figure 3A, Panel 3). We conclude that timely repair of meiotic DSBs in *Mcm5^A7^*mutants requires Lig4-dependent NHEJ.

The most parsimonious interpretation of our γ-H2Av focus data is that there can be both homosynapsis and heterosynapsis within a single nucleus, and that DSBs in regions of homosynapsis are repaired by HR, whereas DSBs in regions of heterosynapsis are repaired by NHEJ. An important question is whether HR is initially attempted at all DSBs, with NHEJ as a backup. To address this question, we determined what proportion of DSBs in *Mcm5^A7^*mutants are dependent on Rad51 (in *Drosophila*, this is encoded by the *spn-A* gene; for simplicity, we refer to the Spn-A protein as Rad51). Meiotic DSBs are not repaired in *spn-A* mutants, resulting in an accumulation of foci between Regions 2A and 3 (Figure 3B). Surprisingly, *Mcm5^A7^ spn-A* double mutants have the same number of γ-H2Av foci in all regions as *spn-A* single mutants (Figure 3B). This implies that all breaks, regardless of whether located in regions of heterosynapsis or homosynapsis, go through the early steps of HR.

### NHEJ-dependent DSB repair in *Mcm5^A7^* mutants is precise

**Figure 4.**
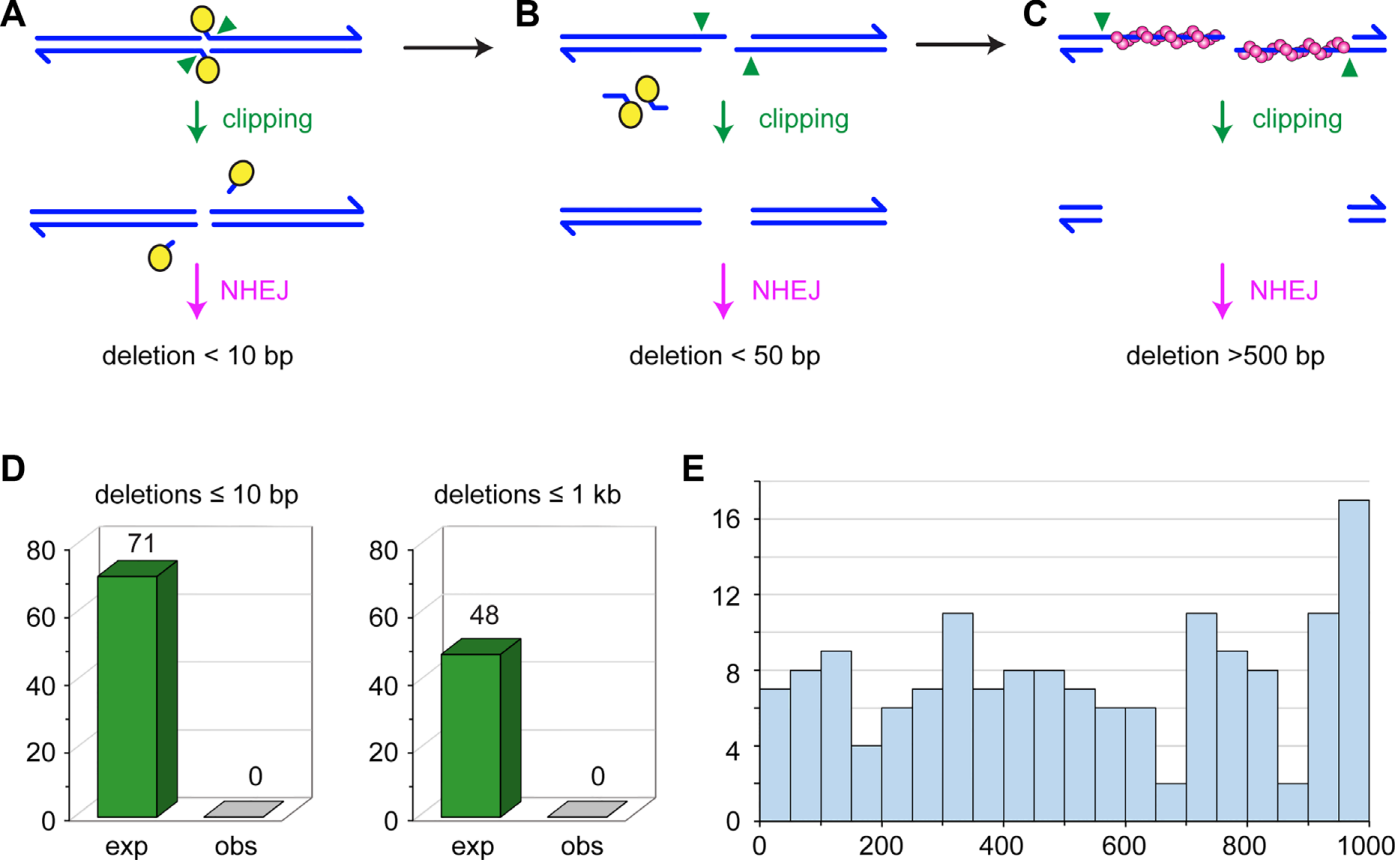
Expected and observed deletions. (**A–C**) Running across the top are structures from canonical models of meiotic recombination. Blues lines represent the two strands of a DNA molecule that is cleaved by Spo11 (yellow ovals). Spo11 remains covalently bound to the two-nucleotide overhanging 5′ ends. These overhangs might be clipped (green arrowheads) to produce ends on which NHJE can act. The repaired chromatid would have a deletion of at least two base pairs, but likely less than ten base pairs. (**B**) During normal resection, Spo11 is released bound to 16–24 nucleotide strands. If this occurs through initial nicks, overhangs of 14–22 nucleotides would be produced, with two base pairs lost. It may be possible that such overhangs could be joined by NHEJ, possibly with additional deletion, but it is likely that clipping would be required to produce a NHEJ substrate; this would result in larger deletions. (**C**) After long resection, Rad51 (magenta spheres) is loaded onto the ends. Given the requirement for Rad51/Spn-A in our experiments, this stage is likely reached at all DSBs. If homology search is unsuccessful, this long overhangs could be clipped to produce a substrate amenable to NHEJ, but the result would be a deletion that, based on gene conversion tract lengths, is would likely be more than 500 base pairs. (**D**) Expected and observed deletions. Based on detection efficiency in simulations, we expected to detect 71 deletions in our dataset if deletions are less than ten base pairs, and 48 deletions if they are less than 1000 bp. We detected zero deletions of either class. (**E**) Histogram of sizes of deletions detected in simulations. Each bin spans 50 bp.

There are several steps in the meiotic recombination pathway at which NHEJ might be enabled. Meiotic DSBs are formed when Spo11 (Mei-W68 in *Drosophila*; for simplicity, we refer to the Mei-W68 protein as Spo11) cleaves with 2-bp 5′ overhanging ends to which the enzyme remains covalently bound (51) (Figure 4A). It is thought that Spo11 blocks binding of Ku, the first step in NHEJ (3,4). Clipping of the 2-nt overhangs by an endonuclease would remove this block but subsequent repair by NHEJ would then result in a deletion of at least two base pairs (Figure 4A). In most models, Spo11-bound oligonucleotides are released during resection (Figure 4B). NHEJ cannot function on long ssDNA overhangs. For example, the 17-nt overhangs generated during excision of *P* transposable elements appear to be refractory to NHEJ (52). These overhangs might also be clipped by and endonuclease (or possibly degraded by an exonuclease); NHEJ would then result in larger deletions, perhaps in the 50–100 bp range (Figure 4B). Finally, for NHEJ to function after long resection the ssDNA regions would need to be removed, and this would generate large deletions (Figure 4C). We do not know the length of resection in *Drosophila*, but average gene conversion tract length of 350–450 bp (42,53,54) suggest a lower limit in this range, or perhaps twice this size if conversions reflects resection on only one side of the break and resection is symmetric. The requirement for Rad51 to repair all DSBs suggests that resection does indeed occur.

Using a conservative estimate of the total number of DSBs in *Drosophila* meiosis, our dataset of whole-genome sequence from 28 progeny of *Mcm5^A7^* mutants described above is expected to include at least 84 sites repaired by NHEJ (see Materials and Methods for calculation details). Strikingly, we did not detect any deletions (Figure 4D). We used several approaches to demonstrate the ability of the methods we used to detect deletions in whole-genome sequence. First, the methods we used were previously used to analyze progeny of a *Drosophila* synaptonemal complex mutant, where several *de novo* deletions were successfully identified (36). For small deletions (<20 bp), we used the Integrative Genomics Viewer (IGV, 33) to compare visual analysis of aligned sequences to variants in the variant call file. This suggested a sensitivity of 89% for detecting insertions and deletions under 40 bp (see Materials and Methods; Supplementary Table S1). We also generated 100 artificial diploid G2 genomes that each had 5–25 randomly placed single-chromatid deletions of 1–20 bp, then simulated Illumina sequencing on one simulated offspring from each genome. 85% of these deletions were detected with the approaches we used. Thus, if NHEJ generates only small deletions, the approaches we used were sensitive enough to detect at least 71 *de novo* deletions.

We assessed our ability to detect large deletions in three ways. First, we examined existing Illumina sequence of flies carrying the *dpp^d-ho^* mutation (55). This deletion was previously estimated from Southern blots to be 2.7 kb (56). The approaches we used detect this deletion and show that it is 3067 bp (2L:2,482,532–2,485,589), which we confirmed by Sanger sequencing of a PCR product. Second, we used IGV to scan for large deletions on the left end of the *w^1118^* parental chromosome and the *X* from four progeny flies that inherited this chromosome (see Materials and Methods). Among the first 20 deletions (mean size of 6554 bp, range 441–10,117 bp), 94 of 100 instances among the five genomes were successfully detected by Pindel, with a mean of 16 supporting sequence reads per deletion (Supplementary Table S2). Third, we repeated the simulations described above, but with deletions up to 1000 bp. We detected 65% of these, across the entire size range (Figure 4E). Based on these results, if the use of NHEJ in repairing meiotic DSBs that cannot find a homolog results in larger deletions, we expect to detect at least 48 deletions in our whole-genome sequence (Figure 4D).

Given our failure to detect any deletions, we considered the possibility of non-allelic homologous recombination or NHEJ resulting in structural rearrangements. We used the same methods used previously to elucidate the multiple inversions of *Drosophila* balancer chromosomes (57,58), but we did not detect any such rearrangements. Taken together, we conclude that when NHEJ is used to repair meiotic DSBs made in the absence of an available homolog, repair is error-free.

## DISCUSSION

Previous studies have found that meiotic DSBs can be repaired by NHEJ when homologous recombination is disrupted at an early step through loss of proteins required to catalyze resection (5–8). Here, we have shown that most DSBs made in *Drosophila Mcm5^A7^* mutants require both Rad51 and Lig4, indicating that NHEJ can be used to repair DSBs even after resection, presumably at sites where the homologous chromosome is not available as a template. Although NHEJ is generally characterized as error-prone, in this situation it is precise.

**Figure 5.**
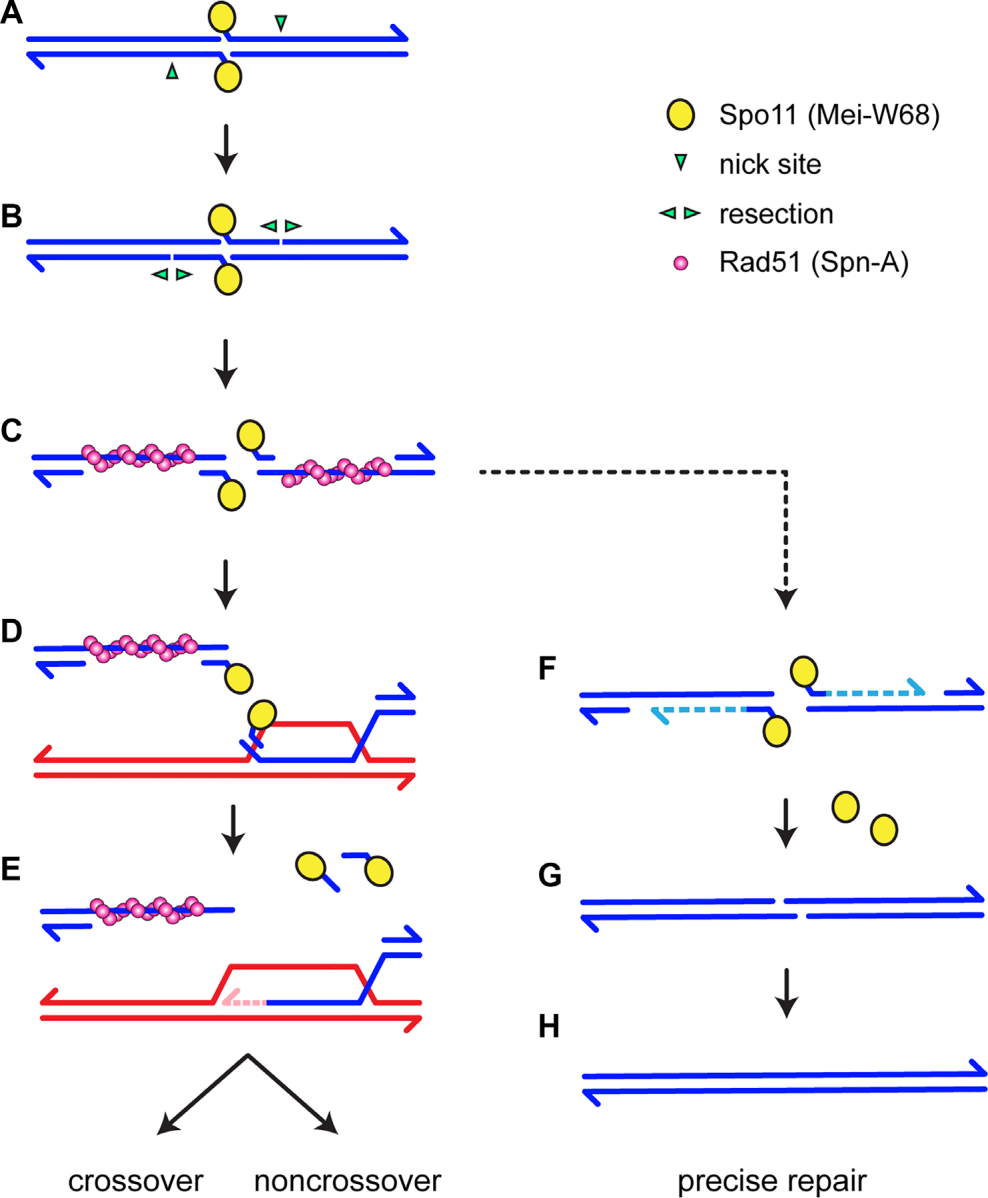
Models for Rad51-, Lig4-dependent meiotic DSB repair. See Supplementary Figure S1 for additional models. In the canonical model (left), DSB formation involves concerted nicking by two Spo11 proteins (yellow circles), which remain covalently bound to 2-nt 5′ overhangs through a tyrosyl phosphodiesterase bond (**A**). Resection is initiated by nicks (green arrowheads) 3′ of the DSB, then (**B**) extended bidirectionally (3). Rad51 and associated proteins (the meiosis-specific Rad51 paralog Dmc1 is not shown because it is not present in *Drosophila*) assembles on the single-stranded DNA exposed by resection (**C**). Recent observations suggest that a short Spo11-oligonucleotide remains annealed at the end of each 3′ overhanging strand (24,25). A homology search and strand exchange produces a D-loop (**D**). Removal of the Spo11-oligonucleotides, either by cleavage or dissociation, allows repair synthesis to proceed (**E**). Subsequent steps (not shown) produce crossover and noncrossovers. We hypothesize that if a homologous template cannot be found, such as in regions of heterosynapsis in *Mcm5^A7^* mutants, the Spo11-oligonucleotides function as primers for fill-in synthesis (**F**). NHEJ can then complete repair through annealing of the 2-nt overhangs (**G**), resulting in precise repair (**H**).

We propose a model for precise post-resection NHEJ based on the recent reports that Spo11-oligonucleotides remain annealed to DSB ends after resection in mouse meiosis (24,25) (Figure 5; Supplementary Figure S1). We hypothesize that Spo11-oligonucleotides remain annealed during the homology search in *Drosophila* (Figure 5A–C). If a homologous template is found, strand exchange produces a D-loop (Figure 5D), after which the Spo11-oligonucleotides are released by dissociation, cleavage of the invading strand to remove the double-stranded region, or enzymatic reversal of the tyrosyl phosphodiesterase bond. This allows repair synthesis to proceed (Figure 5E). We hypothesize that if homology cannot be found, such as in regions of heterosynapsis in *Mcm5^A7^* mutants, the Spo11-oligonucleotides are used as primers for fill-in synthesis (Figure 5F). Spo11 is then enzymatically removed and NHEJ proceeds through annealing of the 2-nt overhangs (Figure 5G) to restore the original sequence (Figure 5H).

A key unknown in our model is how Spo11 is removed. Likely candidates include tyrosyl-phosphodiesterases (TDPs), which directly reverse protein tyrosyl-DNA complexes generated by topoisomerases (reviewed in 59), and proteins with an SprT metalloprotease domain that can remove DNA-protein crosslinks (60–62). The only TDP in *Drosophila* is Tdp1, encoded by the gene *glaikit* (*glk*) (63,64). Because *glk* is an essential gene, we generated flies with germline expression of a transgene for RNAi knockdown of *glk* in *Mcm5^A7^* mutants. We did not detect persistence of γ-H2Av foci in these females (Supplementary Figure S2).

*Drosophila* has orthologs of the SprT domain proteins SPRTN and Germ Cell Nuclear Acidic Peptidase (GCNA). The SPRTN ortholog is encoded by *maternal haploid* (*mh*) (65). We constructed *mh Mcm5^A7^* double mutants but did not detect persistence of γ-H2Av foci (Supplementary Figure S2). The GNCA ortholog is difficult to test because mutants have Spo11-independent DSBs that apparently stem from defects in pre-meiotic S phase, as well as germline developmental defects in the ovary (66).

We do not know whether loss of the ability to remove Spo11 by a TDP or protease would lead to persistence of γ-H2Av foci in *Mcm5^A7^* mutants. Blocking this step might allow cleavage of the 2-nt overhang, as Figure 4A. If this is the case, then foci might still go away with normal kinetics due to NHEJ repair; however, there would be a small deletion at every site that is unable to complete HR. This might provide a way to map DSBs in *Drosophila*.

Our experiments reveal the existence of a novel backup mechanism to repair meiotic DSBs when an HR template cannot be found. This involves repair by NHEJ that, even though resection apparently occurs, is precise. We propose that this is possible because of retention of Spo11-bound oligonucleotides at the ends of the DSBs. In male meiosis in mice, only a small fraction of DSB ends appeared to retain binding of Spo11-oligonucleotides (24,25). Although the tools to detect this intermediate in Drosphila do not exist, we speculate that this intermediate may be more prevalent. In contrast to the situation in mammals, in *Drosophila* DSBs re made after synapsis of homologous chromosomes (67,68). In previous studies of *Mcm5^A7^* we proposed that this relationship results in a robust barrier to using the sister chromatid as a repair template (21), but it may also prevent a broad search for a homologous template for repair. If a DSB is made in a region for which a repair template is not available on the synapsed partner, the fill-in/NHEJ model depicted in Figure 5 may be the only means to achieve error-free repair. We are able to detect this pathway in the *Mcm5^A7^* mutant because synapsis is frequently non-homologous (21).

It is difficult to determine whether or how often this mechanism is used in normal meiosis, but our analysis indicates that ∼3% of the reference *X* chromosome is absent from the *w^1118^* chromosome due to more than 100 deletions of 1–10 kb relative to the reference (mostly differences in transposable element insertions). This is consistent with a larger and more comprehensive survey that reported that commonly used laboratory strains differ by an average of >500 transposable element insertions each (69). The whole-genome sequences of meiotic events by Miller *et al.* (42) suggests that DSBs do occur in transposable element sequences, so these heterologies provide opportunity for DSBs to be made in sequences for which there is no homologous sequence at the allelic site. In some cases, these DSBs may be repaired through non-allelic homologous recombination with a nearby copy of the same transposable element (42), but it is reasonable to anticipate that the NHEJ repair mechanism we propose is not infrequently invoked in normal meiosis. The discovery that Spo11-oligos remain annealed in mouse meiosis (24,25), together with results presented here, suggests that this precise NHEJ backup pathway may be conserved in diverse eukaryotes.

## DATA AVAILABILITY

Illumina reads used in this project have been deposited at the National Center for Biotechnology Information (https://www.ncbi.nlm.nih.gov/) under project PRJNA623030. Code used in this project are available at: https://github.com/danrdanny/mcm5-A7.

## Supplementary Material

gkaa1205_Supplemental_File

## Notes

Present address: Talia Hatkevich, Eva Garland Consulting, Research Triangle Park, NC, USA.
