# Supplementary material for: A pathway for error-free non-homologous end joining of resected meiotic double-strand breaks
Source: Nucleic Acids Res. 2021 Jan 6;49(2):879–90. doi: 10.1093/nar/gkaa1205 (PMC7826270; doi:10.1093/nar/gkaa1205)
Supplement: gkaa1205_Supplemental_File [file gkaa1205_supplemental_file.pdf]

Supplementary data for

A pathway for error-free non-homologous end joining of resected meiotic double-strand breaks

Talia Hatkevich, Danny E. Miller, Carolyn A. Turcotte, Margaret C. Miller, Jeff Sekelsky

corresponding author: Jeff Sekelsky
Email: sekelsky@unc.edu

This PDF file includes:

Tables S1, S2, and S3

Figures S1 and S2

References

Table S1. Detection of small insertions and deletions

Position	Reference	Sample	Q	Size	VCF	IGV
14,872,733	ATATATATATGTATATATAT	ATATATATAT	225	-10		
14,873,143	AAAAAAAAAAAAAAAAAAA	AAAAAAAAAAAAAAAAAA		-1	X	
14,874,478	ATTTTTTTT	ATTTTTTTTTT	142	2		
14,874,548	GGT	GGGT		1	X	
14,874,552	TCG	T	84	-2		
14,880,683	GAAAAAAAAAAA	GAAAAAAAAAAAAA	23	2		
14,882,179	GTTTTTT	GTTTTTTTTT	225	3		
14,882,272	TTCT	TT	79	-2		
14,884,296	TGG	TGGG	22	1		
14,885,840	T	TCA	19	2		
14,885,898	T	TATATGCA	30	7		
14,885,929	TAA	TAAA	116	1		
14,885,977	CTTT	CTT	144	-1		
14,886,089	CAAAAAA	CAAAAAAA	114	1		
14,886,140	TAAAAAAAAAAA	TAAAAAAAA	181	-3		
14,887,936	ATT	AT	136	-1		
14,888,006	CTACTCTTCTAATATTTGTACA	C	225	-21		
14,888,641	GTTTTTTTTTTTTTTTT	GTTTTTTTTTTTTTT	18	-2		
14,889,122	AATTGCA	AA	225	-5		
14,889,243	GAAAAAAA	GAAAAA	147	-2		
14,889,302	AG	A	225	-1		
14,889,388	A	AAACACTGAGTTTGAAT	225	16		
14,889,482	CAAAAAAAAAA	CAAAAAAA	210	-3		
14,889,964	CAAA	CAA	28	-1		
14,889,971	TT	TTTTACTTGGCTAACATGGCGT	225	20		
14,890,405	TCCC	TCCCC	223	1		
14,890,753	GAAAAAAA	GAAAAAA	83	-1		
14,890,902	GTTTTTTTTT	GTTTTTTTTTTTTT	225	4		
14,891,051	TAAAAAAAAAA	TAAAAAAAAAAAA	66	2		
14,891,249	TTTTTCTTTT	TTTTT	225	-5		
14,891,966	TTTATTATTATTATT	TTTATTATTATT	22	-3		
14,891,976	TTAT	TT	222	-2		

Table S1. Detection of small insertions and deletions

Position	Reference	Sample	Q	Size	VCF	IGV
14,892,094	TAAAA	TAA	225	-2		
14,892,154	T	TC	225	1		
14,892,218	TG	T	174	-1		
14,892,284	TA	TAA	225	1		
14,892,413	TGTTAACAAAAAAGGGTT	TGTT	154	-14		
14,892,466	TTTTTTTTTTCC	TTTTTTTTTC		-2	X	
14,893,492	CAAAAAAAAAAAAAAAAAAA	CAAAAAAAAAAAAAAAAA	59	-2		
14,893,782	GAAAAAAAAAA	GAAAAAAAA	115	-2		
14,894,269	AAAACAAA	AAAA	43	-4		
14,894,661	AAAAA	AAAAAAACAAAA	225	7		
14,894,726	GT	GTT	184	1		
14,894,799	AAAGAT	AAAGATACGAGATCCAAGAT	225	14		
14,894,972	TGC	T	120	-2		
14,895,249	T	TC		1	X	
14,895,252	CAT	CATAT	167	2		
14,895,427	CAAAAAAAAAAAAA	CAAAAAAAAAAAA	14	-1		
9,324,670	CTTTTTTTTTT	CTTTTTTTTTTTTT	5	3		
9,325,085	GCCC	GCCCC	222	1		
9,326,382	CC	CCTC	30	2		
9,326,806	AG	AGAAATACACATAAATACATGGGGACG	119	25		X
9,327,114	AGATGGTGTTG	A	222	-10		
9,327,767	GCTTTCCGCACTTTCCGCACTTTCCGC	GCTTTCCGCACTTTCCGCACTTTCCGCA CTTTCCGCACTTTCCGC	222	18		
9,328,675	CCT	C	178	-2		
9,329,897	CAGCATCCAGCACCCAGCATCTAG	CAG	222	-21		
9,333,342	CGGGGCCATCGATGTGGG	CGG	222	-15		
9,333,856	CTTTTTTTTTT	CTTTTTTTTTTTT		2	X	
9,333,988	TAAAAAAA	TAAAAAAAA	30	1		
9,336,316	TTGCT	TT	58	-3		
9,336,819	A	AT	18	1		
9,337,738	GTCTGTTCTG	GTCTGTTCTGTTCTG	222	5		
9,339,328	GGG	GGGTGG	222	3		
9,341,344	T	TGG	13	2		X

Table S1. Detection of small insertions and deletions

Position	Reference	Sample	Q	Size	VCF	IGV
9,341,694	ATTTTTTTT	ATTTTTTT	9	-1		
9,341,831	GCACAAGTACCCTAATACTGGGTAC	GGTACTTGTGGTACCG		-9	X	
9,344,519	CGG	CGGTTGGTTGGGTGGG	222	13		
9,344,917	T	TG	110	1		
9,345,347	TTTTT	TTTTTGTTTT	10	5		
9,347,115	CTCGGG	CTCGGGGTTCGGG	222	7		
9,347,717	CAA	CAAA	222	1		
9,348,643	GTTTTT	GTTTTTT	58	1		
1,469,116	GGTTGCA	G	222	-6		
1,469,227	A	ATT	222	2		
1,469,553	A	AC	0	1	X	
1,469,558	A	ACAAAG	134	5		
1,469,877	TAAA	TAAAA	203	1		
1,469,960	CAA	CAAA	0	1	X	
1,470,009	ATCCAATCCAATCCATTCCAATCCAATCCA	ATCCAATCCAATCCA	221	-15		
1,470,200	CTGCGAGTGCGAGTGCCAGTGC	CTGC	222	-18		
1,470,612	CCAGAATCAGAATCAGAATC	CCAGAATCAGAATC	222	-6		
1,470,707	ATCGAACAGAAAGACCTC	ATC	222	-15		
1,470,999	ACAT	ACATTCTTCGAGAACTTTGCCAT	222	19		
1,471,951	CTTTGTCAGAGAGTT	CTT	222	-12		
1,472,787	CACACACACACATACACACACAC	CACACACACAC	222	-12		
1,473,317	CTATTATT	CTATT	222	-3		
1,473,532	CAAAAAAAAAA	CAAAAAAAAA	0	-1	X	
1,474,530	C	CCCCATT	222	6		
1,474,649	ACAT	A	222	-3		
1,475,256	CAAAAAAAAAA	CAAAAAAAAA	4	-1		
1,477,302	GGAGGTTTAATCAGCAATAG	GG	222	-18		
1,477,441	AACACCACTTCGGGAGTAAGC AATATAACAAAACATTTAC	AAC	222	-37		
1,478,058	AA	AAGA	222	2		
1,479,650	AATTGGGATTGGGATTGGGAT	AATTGGGATTGGGAT	222	-6		
1,479,709	CGAGGAGTGAGG	CGAGG	222	-7		
1,479,767	TCTCGCTCGCTCGCTC	TCTCGCTCGCTCGCTCGCTCGCTC	222	8		

Table S1. Detection of small insertions and deletions

Position	Reference	Sample	Q	Size	VCF	IGV
1,480,119	CGGGGG	CGGGG	84	-1		
1,480,828	TGGAGGAGC	TGGAGGAGCTGGGGAGGAGC	222	11		
1,481,042	GTTTTTTTTTTTTTT	GTTTTTTTTTTTTTTTTT,GTTTTTTTTTTTTTTTTTTT	41	23		
1,481,957	GACAAAAAAAAA	GA	222	-10		
1,481,959	CAAAAAAAAAAAAAAAAAAAAA	CAAAAAAAAAAA,CAAAAAAAAAA	194	2		
1,482,233	GCGTGTCCCGTGTCCCGTGTCCCGTGTCC	GCGTGTCCCGTGTCCCGTGTCC	222	-7		
1,482,489	CATATA	CATA	192	-2		
1,482,526	CGA	CGATTTGGA	222	6		
1,482,620	CGT	C	121	-2		
1,483,609	AATC	AATCAATC	0	4	X	
1,483,615	A	AGGTGT	0	5	X	
1,484,557	GGTCTGCCAGCGTCTGCCAGC	GGTCTGCCAGC	222	-10		
1,484,806	CCCACCACCACCACCACCAC	CCCACCACCACCACCAC	222	-3		
1,484,890	A	ACGCCAAAGACAAGGTCAGGGCGCTGAC	148	27		
1,485,581	CGAGAGAGAGAGAGAGAGAGAGAGAGA	CGAGAGAGAGAGAGAGAGAGAGA	222	-4		
1,487,334	ATCGATGTCCTTGGTA	ATCCTCGATTTGG	0	-3	X	
1,487,438	AAGTAAG	AAGTAAGATTAGTAAG	224	9		
1,488,968	A	ATATAGAG	0	7	X	

A 25 kb segment was selected for each of three different chromosome arms from three different sequenced flies:

top:	fly 02	chrX	14,870,514 - 14,895,513	
middle:	fly 12	chr2R	9,324,228 - 9,349,227	
bottom:	fly 03	chr3L	1,469,051 - 1,494,050	

Insertions and deletions within each region were extracted from the variant call file and compared to visual analysis with the Integrative Genomics Viewer (1). Table lists position of each variant, sequence of the reference and sample, VCF quality score (Q, 1-255; 0 means no call present in the vcf file), size (negative values are deletions relative to the reference, positive values are insertions), and presence in VCF file and by IGV visual analysis (X indicates not present). Among these 114 variant positions there were 13 (11%) false negatives (seen in IGV but not present in the vcf file) and 2 (2%) false positives (present in the vcf file but not in IGV; in both cases there were two reads that supported the variant call but multiple reads that did not. fly 02 was selected for the X chromosome region because that fly inherited the non-reference chromosome. We examined the same region from a fly (05) that inherited the reference chromosome. No variants were detected in this sequence.

Table S2: Detection of large deletions.

Position	Size (bp)	w1118	-02	-06	-16	-24	Reference Genome
318,520	5033	18	13	18	7	18	*flea{}3* in intron of *CG32816*
428,474	6132	20	22	3	16	30	*3S18{}4* in intron of *CG32816*
551,472	7509	16	20	14	15	9	*412{}5* in intron of *Appl*
812,316	5578	14	6	6	12	19	*297{}6* in intron of *CG43867*
827,651	7739	15	13	19	15	13	*roo{}7* in intron of *CG43867*
906,753	1736	16	14	29	20	18	*S{}8* in intron of *CG43867*
1,033,401	7580	13	17	11	14	9	*412{}12* in 5'UTR of *su(w^a)*
1,063,805	8740	16	0	0	8	10	*roo{}13* in intron of *CR44779*
1,357,823	10117	21	22	24	24	16	*Ivk{Doc{}15}1704*
1,518,086	5349	16	11	9	0	6	intergenic
1,567,700	1744	6	9	0	9	12	*I{}18* in intron of *Mur28*
1,628,024	7415	12	0	13	14	8	*gtwin{}19* in intron of *Mur2B*
1,737,160	9112	20	32	42	30	36	*roo{}20* in intron of *Scgdelta*
1,930,587	7518	22	9	0	27	14	*opus{}21*
1,954,936	7887	27	12	23	22	19	*Stalker2{}22* in intron of *Hr4*
2,308,843	8530	15	20	16	24	11	*Tirant{}24* in intron of *Raf*
2,329,221	441	21	14	22	22	25	intron of *Raf*
2,399,990	8295	0	0	0	0	0	*roo{}25*
2,610,435	7064	13	6	12	13	12	*HMS-Beagle{}26*
2,823,836	8495	14	15	6	7	13	*roo{}28* in intron of *kirre*
2,844,949	7357	16	18	21	13	11	*mdg1{}29* in intron of *kirre*
mean:	6554	17	15	17	16	15	mean across all sequences: 16
	n:	20	18	17	19	20	total detections: 94 of 100 (105)

Table S2: Detection of large deletions. The Integrative Genomics Viewer (1) was used to scan for deletions near the left end of the *X* chromosome (chrX) in *w^{1118}* parental fly sequence and the sequences from four progeny (-02, -06, -16, and -24) that inherited a derivative of this chromosome. Sequences to left of position 300,000 were not examined due to poor mapability in the repeat-rich sub-telomeric region. Table lists the first 21 large deletions relative to the reference genome; all were visible in all five sequences. This included one intergenic deletion, one intronic deletion, and 19 transposable elements that are in the reference genome but not on these chromosomes. We used pindel v0.2.5b9 (2) to find deletions in Illumina sequence. Table lists the number of supporting reads, which are those that span the deletion with at least 30 nucleotides on each side. Red denotes cases that were not detected. The *roo{}25* site was not detected in any of these sequences; it may be a complex rearrangement.

Table S3. Meiotic crossovers on chromosome *2L*.

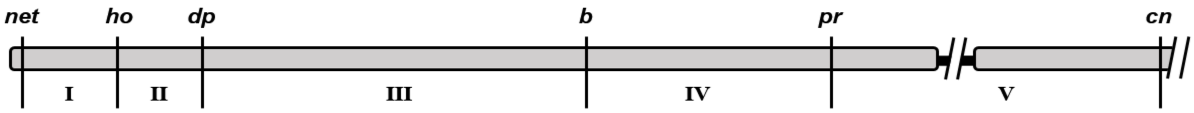

Progeny Class		Maternal Genotype	
		wild type	***Mcm5^{A7}***
Parental		2376	1829
Single crossover (interval)	I	176	4
	II	290	14
	III	1099	16
	IV	154	135
	V	39	6
Double crossover (intervals)	I / II	1	0
	I / III	11	0
	I / IV	10	0
	I / V	2	0
	II / III	6	1
	II / IV	7	2
	II / V	13	0
	III / IV	19	7
	III / V	17	1
	IV / V	2	2
	Totals:	1903	2070

Table S3. Meiotic crossovers on chromosome *2L*. Each row lists the number of total progeny from parental, single crossover, and double crossover classes for wild-type and *Mcm5A7* mutant females. Wild-type data are from (3).

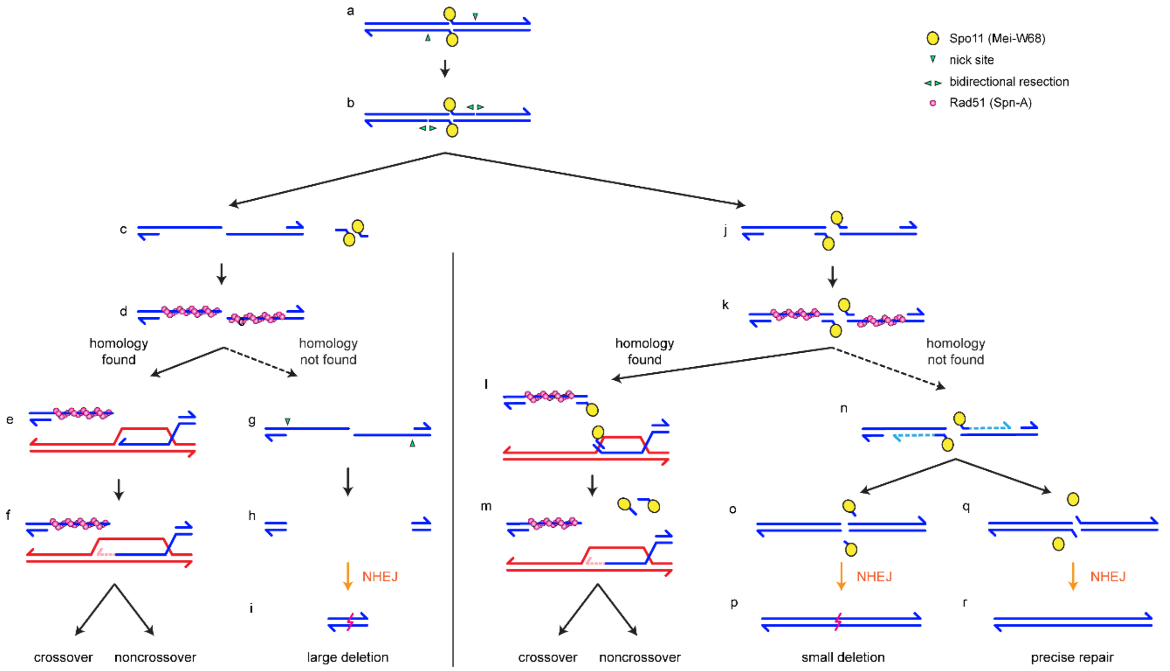

Figure S1. Models for post-resection repair of meiotic DSBs by NHEJ. DSB formation by Spo11 (a) is followed by nicking (b). In most models (left), bidirectional resection that leads to release of Spo11-bound oligonucleotides (c). Rad51 and related proteins are loaded onto the ssDNA (d) and a homology search is done. After a successful homology search, strand exchange generates a D-loop (e) that is extended by synthesis (f). Further steps (not shown) generate crossover or noncrossover products. If a homologous recombination partner is not found (dashed arrow), the overhangs must be nicked (g, green arrowhead), leading to a large gap (h). The ends can now be joined by canonical NHEJ, resulting in a deletion likely to span 100s of base pairs (i). Models on the right are based on recent observations that suggest that Spo11-oligonucleotides remain bound to the 3’ ends after resection (j). The Rad51 filament then spans a region of ssDNA gap (k). Successful strand exchange does not involve the extreme 3’ end (l), but release of the Spo11-oligonucleotides by dissociation or cleavage would allow repair synthesis (m). As in the standard model, further steps lead to crossover or noncrossover products. If the homology search fails (dashed line), the Spo11-bound oligonucleotide could serve as a primer for gap-filling synthesis (n). Removal of Spo11 can be accomplished by clipping the 2-nt overhangs (o). In this case, NHEJ will result in a small deletion (p). Alternatively, Spo11 can be removed by reversal of the tyrosyl phosphodiesterase bond (q). Repair by NHEJ using the complementary 2-nt overhangs would restore the original sequence precisely (r). Our failure to detect either small or large deletions under conditions where NHJE is completing repair (*i.e.*, in the *Mcm5*^{A7} mutant) , is most consistent with this last model.

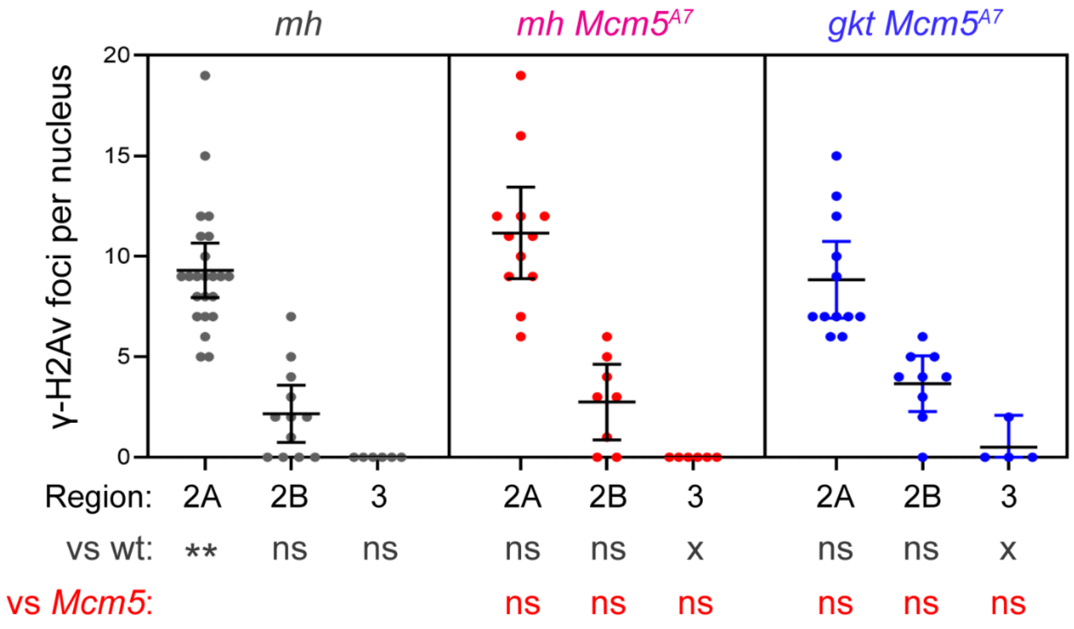

Fig. S2. Meiotic DSB repair in *Mcm5*^{A7} mutants with potential Spo11 nucleases compromised. Each dot is the quantification of γ-H2Av foci in one nucleus; bars show mean and 95% confidence intervals. Results of unpaired *t* tests are shown below: ns, $p > 0.05$, ** $p < 0.01$, x, test cannot be done because all values are 0. n = (for the three regions) for *mh*: 23, 19, 6; for *mh Mcm5*^{A7}: 12, 8, 6; for *gkt Mcm5*^{A7}: 12, 10, 4. The genotype listed as *mh* is *mh*; [*Mcm5*^{A7} or *Df(3R)Exel7305*] / *TM6B*. The *gkt* in the genotype above is a combination of the RNAi transgene *TRiP.HMJ24083* (4) and *nos::GAL4*, which expresses before meiosis begins and throughout pachytene. In the absence of an antibody, we were unable to verify knockdown of GKT protein.

Supplemental References

1. Robinson, J.T., Thorvaldsdottir, H., Wenger, A.M., Zehir, A. and Mesirov, J.P. (2017) Variant review with the Integrative Genomics Viewer. *Cancer Res.*, **77**, e31-e34.
2. Ye, K., Schulz, M.H., Long, Q., Apweiler, R. and Ning, Z. (2009) Pindel: a pattern growth approach to detect break points of large deletions and medium sized insertions from paired-end short reads. *Bioinformatics*, **25**, 2865-2871.
3. Hatkevich, T., Kohl, K.P., McMahan, S., Hartmann, M.A., Williams, A.M. and Sekelsky, J. (2017) Bloom syndrome helicase promotes meiotic crossover patterning and homolog disjunction. *Curr. Biol.*, **27**, 96-102.
4. Ni, J.Q., Zhou, R., Czech, B., Liu, L.P., Holderbaum, L., Yang-Zhou, D., Shim, H.S., Tao, R., Handler, D., Karpowicz, P. *et al.* (2011) A genome-scale shRNA resource for transgenic RNAi in Drosophila. *Nat. Methods*, **8**, 405-407.
